# Sarcopenia interventions in long‐term care facilities targeting sedentary behaviour and physical inactivity: A systematic review

**DOI:** 10.1002/jcsm.13576

**Published:** 2024-09-18

**Authors:** Yihan Mo, Linghui Chen, Yuxin Zhou, Anna Bone, Matthew Maddocks, Catherine J. Evans

**Affiliations:** ^1^ Cicely Saunders Institute of Palliative Care, Policy and Rehabilitation, Florence Nightingale Faculty of Nursing, Midwifery and Palliative Care King's College London London UK

**Keywords:** intervention, logic model, long‐term care facility, physical inactivity, sarcopenia, sedentary behaviour, systematic review, Theory of Change

## Abstract

**Background:**

Sedentary behaviour and physical inactivity are independent risk factors for sarcopenia for long‐term care facility residents. Understanding the components, mechanisms and context of interventions that target change in these risk factors can help optimize sarcopenia management approaches. This study aimed to identify, appraise and synthesize the interventions targeting sedentary behaviour and physical inactivity, construct a Theory of Change logic model, inform complex sarcopenia intervention development and identify areas for improvement.

**Methods:**

Eight electronic databases, including Embase and Web of Science, were searched for eligible interventional studies from inception until February 2024. Narrative synthesis was used. The Theory of Change was applied to develop a logic model presenting the synthesized results. A Cochrane risk of bias assessment tool was used for quality appraisal.

**Results:**

The study included 21 articles involving 1014 participants, with mean ages ranging from 72.5 to 90.4 years. The proportion of female participants ranged from 8.0% to 100.0%. The applied sarcopenia diagnosis criteria varied, including those of the Asian Working Group for Sarcopenia and the European Working Group on Sarcopenia in Older People. The overall risk of bias in the included studies was moderate. Interventions primarily targeted physical inactivity, with resistance training being the most common intervention type. The reporting of intervention adherence was insufficient (only 11 out of 21 included studies provided adherence reports), and adherence overall and by intervention type was not possible to discern due to inconsistent criteria for high adherence across these studies. Four categories of intervention input were identified: educational resources; exercise equipment and accessories; monitoring and tailoring tools; and motivational strategies. Intervention activities fell into five categories: determining the intervention plan; educating; tailoring; organizing, supervising, assisting and motivating; and monitoring. While sarcopenia‐related indicators were commonly used as desired outcomes, intermediate outcomes (i.e., sedentary time and physical activity level) and other long‐term outcomes (i.e., economic outcomes) were less considered. Contextual factors affecting intervention use included participant characteristics (i.e., medical condition and education level) and intervention provider characteristics (i.e., trustworthiness).

**Conclusions:**

The findings led to the development of a novel logic model detailing essential components for interventions aimed at managing sarcopenia in long‐term care facilities, with a focus on addressing sedentary behaviour and physical inactivity. Future sarcopenia interventions in long‐term care facilities should fully attend to sedentary behaviour, enhance adherence to interventions through improved education, monitoring, tailoring and motivation and establish an agreed standard set of outcome measures.

## Introduction

The world's population is aging, with the fastest growing section being those aged 80 years and over.^1^ Aging is the primary driver for most diseases or disorders, including sarcopenia.^2^ Sarcopenia is an age‐related disease characterized by the loss of skeletal muscle mass plus the loss of muscle strength and/or reduced physical performance.^3^ Individuals who suffer from sarcopenia exhibit a decrease in their gait speed, hand grip strength and ability to transfer postures from lying to sitting and standing when compared to those who do not have sarcopenia. Additionally, they are at a greater risk of experiencing disability, morbidity, mortality and diminished quality of life.^4^, ^5^, ^6^, ^7^, ^8^

The global prevalence of sarcopenia ranges from 0.2% to 86.5% using different sarcopenia definitions and different sarcopenia parameter measurement cut‐off points.^9^ Sarcopenia definitions vary with regard to the criteria for diagnosis. Some define sarcopenia as low muscle mass combined with low grip strength and/or slow gait speed (e.g., European Working Group on Sarcopenia in Older People [EWGSOP]^10^ and Asian Working Group for Sarcopenia [AWGS]^11^), while others focus solely on muscle mass and strength (e.g., Revised EWGSOP [EWGSOP2]^12^ and Foundation for the National Institutes of Health [FNIH]^13^). Cut‐off values for these parameters have been tailored to specific populations and validated accordingly, such as AWGS for Asian populations and EWGSOP for European populations. AWGS^11^ and EWGSOP2^12^ also recommend case‐finding methods to identify people at risk of sarcopenia (i.e., merely with low muscle mass, low muscle strength or reduced physical performance). The prevalence of sarcopenia has been reported to be higher for older adults in long‐term care facilities (LTCFs) than in home‐dwelling settings,^14^ and it is expected to rise as the aging population continues to grow.

The co‐occurrence of sedentary behaviour and physical inactivity increases the risk of sarcopenia,^12^, ^15^ attributed to a reduced muscle protein synthetic response to protein and amino acid‐based nutrition.^16^ According to the established definition, sedentary behaviour refers to any activity that involves sitting, reclining or lying down with minimal energy expenditure, equivalent to or less than 1.5 metabolic equivalents of task.^17^, ^18^ In contrast, physical inactivity is characterized by an insufficient amount of moderate‐to‐vigorous physical activity that fails to meet the recommended guidelines for a given age group.^16^, ^19^ According to reports, older adults residing in LTCFs engage in sedentary activities for approximately 85% of their waking hours^16^ and exhibit greater levels of physical inactivity compared to their counterparts living in the community.^20^ Interventions targeting sedentary behaviour and physical inactivity have been considered in trials to reduce sarcopenia. For example, for both home‐dwelling and LTCF residents, multicomponent training involving aerobic exercise, strength resistance exercise and balance training has demonstrated effectiveness in reducing sarcopenia.^21^, ^22^, ^23^, ^24^ Although several interventions^25^, ^26^, ^27^ have been designed to reduce sedentary behaviour and increase physical activity levels among LTCF residents, there is a lack of detailed descriptions regarding the development phase or use of theory to inform the creation or evaluation of complex sarcopenia interventions in LTCFs. Due to the specificity in terms of the complex conditions and needs of LTCF residents (e.g., physical capabilities, medical comorbidities and symptom burdens)^28^ and the complex context of LTCFs,^29^ it is necessary to understand how and why these interventions work for LTCF residents and how these interventions could be adapted for this setting.

The objectives of this study are to (1) identify, appraise and synthesize the published evidence on interventions targeting sedentary behaviour and physical inactivity in LTCFs for sarcopenia; (2) construct a Theory of Change logic model with the findings to inform complex sarcopenia intervention development; and (3) identify areas for improvement and propose recommendations.

## Methods

### Study design

The protocol of this systematic review was prepared in accordance with the Preferred Reporting Items for Systematic Reviews and Meta‐Analyses (PRISMA) protocol 2020 statement.^30^ The protocol was registered on the PROSPERO database (CRD42023394385).

### Theoretical underpinning

The Theory of Change^31^ forms the basis of the systematic review and logic model, serving as a pragmatic framework to illustrate how interventions drive change.^32^ It enables researchers to articulate the issues they aim to address, desired changes and planned actions.^33^ Logic models, on the other hand, function as comprehensive tools for planning, managing and evaluating interventions by systematically outlining the components influencing change and their interconnections.^34^ This aids in conceptualizing complex review questions.^35^ Visually, logic models illustrate the relationships between intervention activities and desired outcomes, outlining underlying assumptions and contextual factors.^34^ They also detail the change mechanisms and factors that may moderate or mediate outcomes.^36^

### Search strategy and eligibility criteria

Electronic searches were undertaken across eight bibliographic databases (English databases: MEDLINE, Cochrane Library, Embase, Web of Science, APA PsycInfo and CINAHL, and Chinese databases: CNKI and Wanfang) to search published evidence on exercise interventions for LTCF residents. We applied MeSH terms and text words encompassing ‘sarcopenia’, ‘exercise’, ‘long‐term care facilities’ and ‘aged’. Databases have been searched from inception until 26 February 2024 with no restrictions on publication date or language. Detailed search strategies for each database are shown in *Tables* *S1*. Electronic searches were supplemented by checking the reference list of included studies and by consulting experts for recommendations to identify potentially eligible studies. We required full journal publications. If these were unavailable or lacked sufficient information or data for extraction, we attempted to obtain the necessary data by contacting the authors.

The eligibility criteria for articles comprised the following: (1) Study types are intervention studies with a comparator (e.g., randomized control trials [RCTs], quasi‐experimental trials and pilots) or intervention studies with no comparators (e.g., single‐arm pretest–posttest intervention trials); (2) study settings are LTCFs (e.g., residential care homes and nursing homes); (3) participants are adults at risk of sarcopenia or with sarcopenia defined by widely accepted consensus (e.g., EWGSOP,^10^ AWGS^11^ and Strength, Assistance walking, Rising from a chair, Climbing stairs, and Falls [SARC‐F] questionnaire^37^); and (4) interventions target sedentary behaviour and/or physical inactivity. Studies were excluded if (1) the study type is a conference abstract, opinion piece, editorial or discussion article; (2) the study setting is home‐dwelling or hospital; or (3) participants have neurological conditions that affect muscle health, such as motor neuron disease.

### Data management and selection process

Following the database search, all identified records were imported into Endnote 20.0 software to remove duplications. Then Covidence software (http://www.covidence.org) was used to manage, screen and identify eligible publications based on the eligibility criteria. Screening and selection of articles were reported according to the PRISMA criteria and flow chart. Two reviewers (Y. M. and L. C.) independently screened and reviewed 20% of all titles and abstracts. Regular reviewer meetings were held during this process. After all uncertainties were discussed and agreement reached, one reviewer (Y. M.) screened the remaining 80% of the titles and abstracts independently. Records that appeared to meet the criteria or had any uncertainty were further screened in full text. Full text records were reviewed by both reviewers (Y. M. and L. C.) first and discussed when there was any disagreement. A third reviewer (C. J. E., M. M., A. B. or Y. Z.) was invited when the disagreement was unresolved.

### Risk of bias assessment

The risk of bias of included studies was assessed using the revised Cochrane Risk of Bias (RoB2) tool^38^ for RCTs and the Risk Of Bias In Non‐randomised Studies ‐ of Interventions (ROBINS‐I)^39^ tool for non‐randomized studies (non‐RCTs) separately. For RCTs, the reviewers assessed five domains: the randomization process, deviations from intended interventions, missing outcome data, measurement of outcomes and selection of the reported result. For non‐RCTs, there were seven domains: confounding, selection of participants into the study, classification of interventions, deviations from the intended intervention, missing data, measurement of outcomes and selection of the reported result. All risk of bias assessments were conducted in duplicate by three reviewers (Y. M., L. C. and Y. Z.). Disagreements were resolved by discussing.

### Data extraction

The data from each included article were extracted according to a data collection template. The template was developed and piloted based on the Template for Intervention Description and Replication (TIDieR)^40^ and the Theory of Change guideline.^32^ The first draft of this template included the following information: (1) article title, authors and year of publication; (2) study design; (3) participant description: age, sex, ethnicity and medical conditions; (4) sarcopenia criteria applied; (5) intervention components for sedentary behaviour and physical inactivity (e.g., types, intensity and frequency); (6) delivery process of the interventions for sedentary behaviour and physical inactivity separately (e.g., by who, how and in where); (7) any tailoring methods, personalized assessment or motivating technique of the interventions; (8) outcome measurements; and (9) any factors influencing the interventions. The data were extracted by Y. M. and checked by L. C. A third reviewer, C. J. E., M. M., A. B. or Y. Z., assisted in reaching a consensus in the event of any disagreements. For information or data with limited detail, we presented them to the maximum extent and noted what information was missing.

### Data synthesis

Narrative synthesis was conducted following the Guidance on the Conduct of Narrative synthesis in Systematic Review.^41^ Intervention syntheses were undertaken by summarizing the components and processes of interventions targeting sedentary behaviour and physical inactivity in LTCFs. Using the concept of the Economic, Clinical and Humanistic Outcomes (ECHO) Model,^42^ the reported long‐term outcomes were categorized accordingly. Drawing on the findings, a Theory of Change logic model was first conceptualized and drafted by the first reviewer (Y. M.), then evaluated by the second and third reviewers (L. C. and Y. Z.) and finalized in consensus with all the co‐authors (Y. M., L. C., Y. Z., A. B., M. M. and C. J. E.).

### Development of a logic model

We developed a logic model based on the synthesized results, including processes (inputs, activities and outputs) and outcomes (short‐term, intermediate‐term and long‐term outcomes).^34^ Inputs refer to the resources that go into an intervention; activities are events undertaken by the intervention to achieve outcomes; and outputs mean the direct results of activities. Outcomes are the desired results of the programme. Short‐term outcomes refer to the immediate effects of the activities and could be observed at the same time of intervention implementation (e.g., knowledge and actions).^32^, ^34^ Intermediate‐term outcomes refer to changes or effects that occur as a direct result of the short‐term outcomes and pave the way towards achieving the long‐term goals, such as behaviour, normative and policy changes.^32^, ^34^ Long‐term outcomes are the final results the programme can achieve on its own and take longer to accomplish. Examples include clinical, humanistic and economic outcomes.^32^, ^34^ The time required to achieve the outcomes can vary depending on the specific situation. Assumptions are beliefs about the intervention and the resources involved. Contextual factors describe the environment in which the programme exists and external factors that interact with and influence the intervention.^34^

## Results

### Study retrieval and characteristics

**Figure 1:**
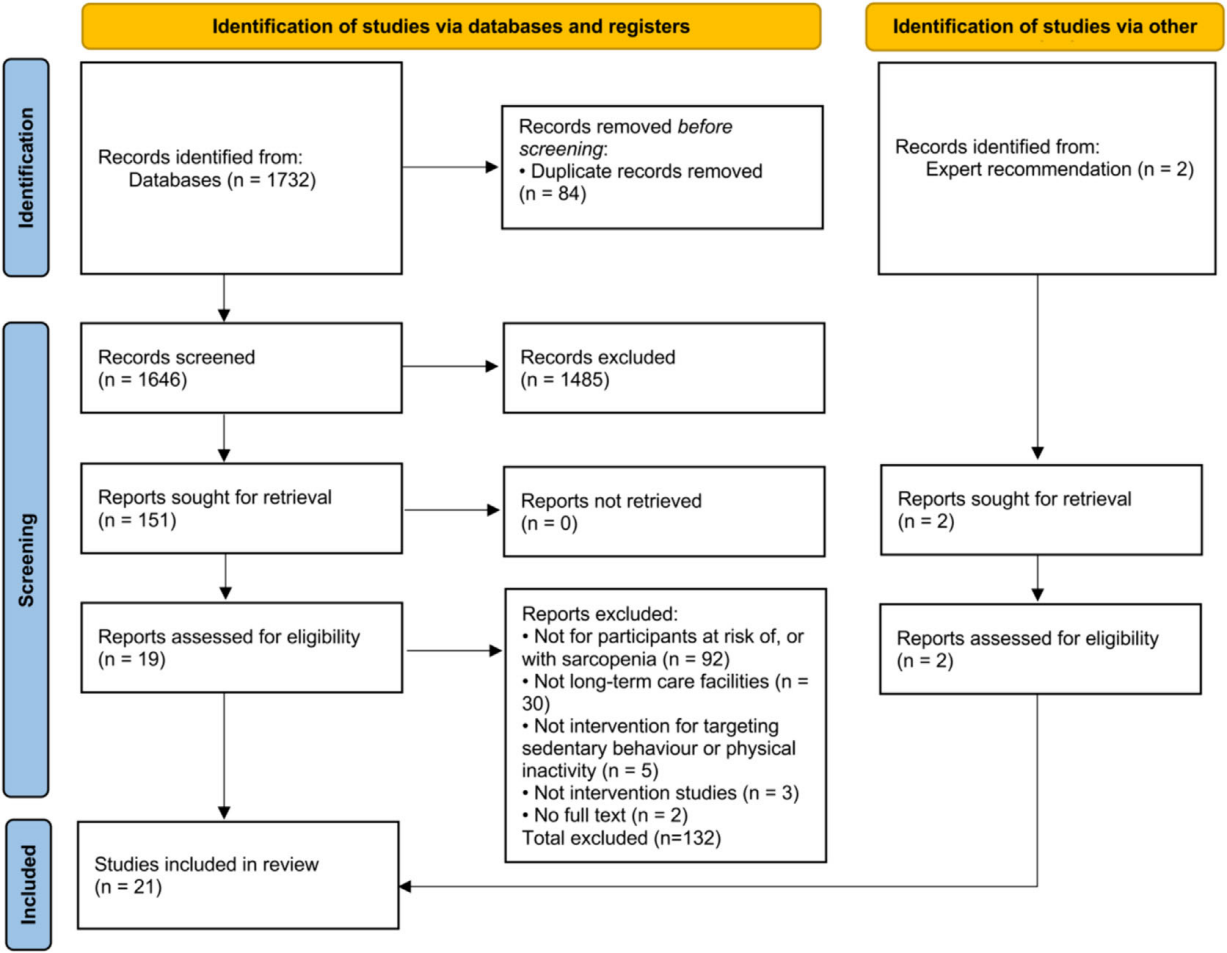
Flow chart for the study selection process.

A total of 1732 articles were identified through an initial literature search. After the removal of duplicates, 1646 articles were further screened for inclusion based on title and abstract. This was followed by a full‐text screening of the 151 articles that remained. The primary cause of ineligibility was not targeting participants at risk of or with sarcopenia. Finally, 21 articles^25^, ^26^, ^27^, ^43^, ^44^, ^45^, ^46^, ^47^, ^48^, ^49^, ^50^, ^51^, ^52^, ^53^, ^54^, ^55^, ^56^, ^57^, ^58^, ^59^, ^60^ were included in this systematic review (*Figure* *1*). The characteristics of the included studies are shown in *Table* *1*. Out of the total studies included, two are in Chinese^45^, ^51^ and others in English.^25^, ^26^, ^27^, ^43^, ^44^, ^46^, ^47^, ^48^, ^49^, ^50^, ^52^, ^53^, ^54^, ^55^, ^56^, ^57^, ^58^, ^59^, ^60^ The type of included studies consists of RCTs (*n* = 8),^46^, ^49^, ^50^, ^52^, ^53^, ^56^, ^57^, ^58^ two‐arm quasi‐experimental trials (*n* = 7)^25^, ^26^, ^27^, ^45^, ^48^, ^51^, ^55^ and single‐arm pretest–posttest interventional trials (*n* = 6).^43^, ^44^, ^47^, ^54^, ^59^, ^60^ These studies were conducted in 11 countries or regions, mainly Taiwan (*n* = 7).^25^, ^26^, ^47^, ^50^, ^53^, ^59^, ^60^ The 21 included articles comprised 1014 participants, with the mean study age ranging from 72.5 to 90.4 years. The proportion of female participants ranged from 8.0% to 100.0%. None of the included studies reported ethnicity. Fifteen studies were undertaken in nursing homes^25^, ^26^, ^44^, ^46^, ^47^, ^48^, ^49^, ^50^, ^52^, ^54^, ^55^, ^56^, ^58^, ^59^, ^60^ and two in residential care facilities with no onsite nursing.^45^, ^51^ The remaining studies stated LTCFs, without further clarification.^27^, ^43^, ^53^, ^57^ The criteria for sarcopenia applied in the included studies varied. AWGS was mostly used (*n* = 7^26^, ^45^, ^48^, ^50^, ^51^, ^53^, ^60^), followed by EWGSOP (*n* = 5^27^, ^43^, ^44^, ^49^, ^55^), FNIH (*n* = 2^54^, ^56^) and the SARC‐F questionnaire (*n* = 1^46^). Additionally, other studies^25^, ^47^, ^52^, ^57^, ^58^, ^59^ only used one to two sarcopenia parameters, each with specific cut‐off points validated for their population.

**Table 1:** Summary characteristics of the included studies

Study	Setting	Sarcopenia criteria applied	Participants	Intervention	Control	Outcomes (intervention group direction of effect)			
						Clinical		Humanistic	Economic
						Sarcopenia related	Other		
Randomized controlled trial									
Chiang et al. (2021) Taiwan	Nursing home	AWGS	• Sample size: IG‐1: 12, IG‐2: 12, CG: 12 • Age (mean years): IG‐1: 85, IG‐2: 85, CG: 84 • Female %: IG‐1: 8, IG‐2: 18, CG: 2 • Conditions: >50% cardiovascular disease, COPD, diabetes, chronic kidney disease, dementia.	Milk combined with low‐intensity resistance exercise training (IG‐1) Soymilk combined with low‐intensity resistance exercise training (IG‐2)	Low‐intensity resistance exercise training	• Muscle mass: Skeletal muscle mass index (+), calf circumference (+) • Muscle strength: Hand grip strength (+) • Physical performance: Gait speed (+) • Per cent with sarcopenia: (−)	• Body weight (+), body mass index (+), body fat (−) • Nutritional index: Prealbumin IG‐1 (+), IG‐2 (−); 25‐hydroxy vitamin D (−) • Inflammation index: C‐reactive protein IG‐1 (+), IG‐2 (−) • Insulin: IG‐1 (=), IG‐2 (+); insulin resistance index IG‐1 and IG‐2 (+)		
Grönstedt et al. (2020) Sweden	Nursing home	SARC‐F questionnaire	• Sample size: IG: 60, CG: 60 • Age (mean years): IG: 86, CG: 86 • Female %: IG: 65, CG: 58 • Conditions: 64% need of walking aid in persons who could walk, mean score of MMSE decreased in both groups.	Sit‐to‐stand (STS) exercises combined with oral nutritional supplement (ONS)	Standard care	• Physical performance: 30‐s chair‐to‐stand test (+), walking speed (−)	• Body weight (+), body mass index (+), fat free mass index (+) • Nutritional index: P‐Albumin (−), vitamin D (−), Mini Nutritional Assessment‐short form score (=) • Inflammation index: High‐sensitivity C‐reactive protein (=) • Functional independence (−), Berg balance scale (−)	• Quality of life: HRQoL, EQ 5D‐5L score (−)	Caregiver time (−)
Courel‐Ibáñez et al. (2021) Spain	Nursing home	FNIH	• Sample size: IG: 12; CG: 12 • Age (mean years): IG: 84; CG: 87 • Female %: Total 58 • Conditions: Not stated.	Vivifrail tailored, multicomponent exercise programme	Same programme with different durations	• Muscle strength: Hand grip strength (+) • Physical performance: Short Physical Performance Battery test score (+), timed‐up‐to‐go test time (−), gait speed (+), sit‐to‐stand test (+)			
Cebrià i Iranzo et al. (2018) Spain	Nursing home	Skeletal Muscle Mass Index plus Gait Speed (with cut‐off points for Spain population)	• Sample size: IG‐1: 27, IG‐2: 27, CG: 27 • Age (mean years): IG‐1: 87, IG‐2: 83, CG: 81 • Female %: IG‐1: 56; IG‐2: 82, CG: 71 • Conditions: >50% cardiovascular, endocrine and neurological disease.	Inspiratory muscle training (IG‐1) versus peripheral muscle training (IG‐2)	Routine daily activities	• Muscle mass: Skeletal muscle mass/body mass index (=) • Muscle strength: Quadriceps femoris strength (+), biceps brachii strength: IG‐1 (−), IG‐2 (+); hand grip strength (+) • Physical performance: Gait speed (=)			
Najafi et al. (2018) Iran	Nursing home	Balance plus Distance walked plus Muscle strength	• Sample size: IG: 35, CG: 35 • Age (mean years): total 73 • Female %: IG: 66, CG: 64 • Conditions: Most have arthritis and a history of using sedative drugs.	Fun physical activities	Regular physical activities	• Muscle strength: Hand grip strength (+) • Physical performance: 6‐min walking test (+)	• Functional balance: Berg balance scale score (+)		
Rogan et al. (2016) Netherland	Long‐term care facility	Short Physical Performance Battery test	• Sample size: IG: 16, CG: 15 • Age (mean years): IG: 90, SG: 87 • Female %: IG: 62, CG: 71 • Conditions: Not stated.	Whole‐body vibration and dance video game	Low‐intensity WBV and stepping exercises	• Physical performance: Short Physical Performance Battery score (+)	• Joint range of motion: Knee extension and flexion (+)		
Tung et al. (2022) Taiwan	Long‐term care facility	AWGS probable sarcopenia	• Sample size: IG: 57; CG: 57 • Age (mean years): IG: 80; CG: 79 • Female %: IG: 53, CG: 58 • Conditions: >90% with chronic diseases.	Vitality Acupunch exercise	Routine daily activities		• Activities of daily living (+) • Body flexibility (+) • Muscular endurance (+) • Joint range of motion (+)		
Urzi et al. (2019) Slovenia	Nursing home	EWGSOP	• Sample size: IG: 18; CG: 17 • Age (mean years): IG: 84; CG: 88 • Female %: Total 100 • Conditions: Approx. 35% sarcopenia; 20% type 2 diabetes; 45% hypertension.	Progressive elastic resistance training programme of moderate intensity	Usual care	• Muscle mass (+) • Muscle strength: Hand grip strength (−) • Physical performance: Gait speed (−), chair‐rise time performance (+), Short Physical Performance Battery score (+)	• Fat mass (−) • Inflammation index: Interleukin‐15 (+), interleukin‐8 (+), C‐reactive protein (−) • Resistin (+), glucose (−) • Brain‐derived neurotrophic factor (+)		
Two‐arm quasi‐experimental trial									
Chang et al. (2020) Taiwan	Nursing home	AWGS	• Sample size: IG: 61; CG: 62 • Age (mean years): Total: 80 • Female %: Not stated • Conditions: Cumulative Illness Rating Scale (points): IG, 2.8; CG, 2.7.	Resistance training	Usual care			• Quality of life: EuroQoL‐5 domains‐3 levels (+) • Self‐perceived physical and mental health (+)	
Chiu et al. (2018) Taiwan	Nursing home	Skeletal Muscle Mass divided by Body Mass (with cut‐off points for Shanghai population)	• Sample size: IG: 33; CG: 31 • Age (mean years): Total: 80 • Female %: Total 50 • Conditions: Cumulative Illness Rating Scale (points): IG, 2.8; CG, 2.7.	Resistance training	Usual care	• Muscle mass: Skeletal muscle mass index (+), skeletal mass per cent (+) • Muscle strength: Grip strength (+), pinch strength (+)	• Body fat per cent (−) • Total functional independence measure score (+), functional independence measure self‐care (+)		
Dong et al. (2021) China	Residential care home	AWGS	• Sample size: IG: 31; CG: 29 • Age (mean years): IG: 77, CG: 79 • Female %: IG: 68, CG: 59 • Conditions: >70% chronic diseases.	Chair‐based resistance training with elastic belt	Usual care	• Muscle mass: Total muscle mass (+), skeletal muscle mass index (+) • Muscle strength: Hand grip strength (+), 5‐time sit‐to‐up test (−) • Physical performance: Timed‐up‐to‐go test (−)	• Functional balance: Berg balance scale (+)		
He et al. (2022) China	Residential care home	AWGS	• Sample size: IG: 13; CG: 13 • Age (mean years): IG: 73, CG: 71 • Female %: Total 31 • Conditions: 50% sarcopenia.	Resistance exercise training with elastic belt to participants with sarcopenia	Same training for residents without sarcopenia	• Muscle mass: Skeletal muscle mass index (+) • Muscle strength: Hand grip strength (+) • Physical performance: Gait speed (+), timed‐up‐to‐go test (−)	• Body weight (−), body mass index (−) • Functional balance: Berg balance scale (−)	• Quality of life: SF‐36 (+)	
Hassan et al. (2016) Australia	Nursing home	EWGSOP	• Sample size: IG: 21; CG: 21 • Age (mean years): IG: 86, CG: 86 • Female %: Not stated • Medical conditions: Not stated.	Progressive resistance and balance training	Usual care	• Muscle mass: Skeletal muscle mass index (+) • Muscle strength: Hand grip strength (+) • Physical performance: Gait speed (+)	• Body mass index (−), body fat (−)		
Tsugawa et al. (2020) Japan	Nursing home	AWGS	• Sample size: IG: 19; CG: 18 • Age (mean years): IG: 83, CG: 86 • Female %: Total 60 • Medical conditions: Charlson comorbidity index: IG: 1.4, CG: 1.5.	Exercise with gymnastics and balls recreation	Usual care	• Muscle mass: Skeletal muscle mass index (−) • Muscle strength: Grip strength (+) • Per cent of sarcopenia (−)	• Activities of daily living: Barthel index (−) • Cognitive function: Mini‐Mental State Examination (+) • Central executive function: Trail Making Test (−)	• Depression: Geriatric Depression Scale‐15 (−)	
Yoshiko et al. (2017) Japan	Long‐term care facility	EWGSOP	• Sample size: IG: 17; CG: 15 • Age (mean years): IG: 78, CG: 73 • Female %: IG: 59, CG: 60 • Conditions: Some with diabetes, hyperlipidaemia or high blood pressure.	Resistance and endurance training	Regular medical checkup	• Muscle mass: Muscle thickness (+) • Muscle strength: Knee extension (+), sit‐to‐stand repetitions (+), hand grip strength (−) • Physical performance: 5‐m walking time (−), timed‐up‐to‐go test time (−) • Sarcopenia prevalence (=)	• Subcutaneous fat thickness (+) • Nutritional index: Protein (−), albumin (+), total cholesterol (+), LDL‐cholesterol (+), HDL‐cholesterol (−) • Leptin (+), insulin (+), tumour necrosis factor‐α (−), adiponectin (+)		
Single‐arm, pretest–posttest design quasi‐experimental trial									
Buendía‐Romero et al. (2020) Spain	Nursing home	FNIH	• Sample size: *n* = 14 • Age (mean years): 82 • Female %: 57 • Medical conditions: 64% with physical frailty or prefrailty status.	Vivifrail tailored, multicomponent exercise programme	—	• Muscle strength: Hand grip strength (+) • Physical performance: 4‐ and 6‐m walking time (−), 5‐repeated sit‐to‐stand test time (−), Short Physical Performance Battery test score (+), timed‐up‐to‐go test (−), SARC‐F score (−)	• Activity of daily living: Barthel index (points) (+), Lawton index (points) (+)		
Chen et al. (2021) Taiwan	Nursing home	AWGS	• Sample size: *n* = 30 • Age (mean years): 75 • Female %: 67 • Conditions: Not stated.	Progressive resistance training rehabilitation via virtual reality	—	• Muscle mass: Skeletal muscle mass index (+) • Muscle strength: Grip strength (+), biceps strength (+), triceps strength (+) • Physical performance: Gait speed (+)	• Fat (free) mass index (+) • Joint range of motion: Shoulder, elbow, wrist flexion and extension (+)		
Chang et al. (2018) Taiwan	Nursing home	Skeletal Muscle Mass Index plus Handgrip Strength (with cut‐off points for Malaysia population)	• Sample size: *n* = 17 • Age (mean years): 82 • Female %: 29 • Conditions: 24% with diabetes, >50% experienced fall(s) and in the prefrail stage.	Whole‐body vibration training	—	• Muscle mass: Skeletal muscle mass index (+) • Muscle strength: Hand grip strength (+) • Physical performance: 5‐repeated sit‐to‐stand test time (−), 8‐foot up and go test (−)	• Joint range of motion: Shoulder–arm flexibility (+) • Balance: Standing on one foot (+)	• Quality of life: EuroQoL‐5 domains‐3 levels and visual analogue (+)	
del Campo et al. (2019) Mexico	Nursing home	EWGSOP	• Sample size: *n* = 17 • Age (mean years): 78 • Female %: 74 • Conditions: Not stated.	Resistance exercise training with dumbbells and elastic belts	—	• Muscle mass: Skeletal muscle mass index (=) • Muscle strength: Grip strength (+) • Physical performance: 4‐m walking time (−), 5‐repeated chair‐stand test time (−), Short Physical Performance Battery test (+)	• Body weight (+), body mass index (+), body fat per cent (+) • Functional balance: Balance test (+)		
Dimori et al. (2018) Italy	Long‐term care facility	EWGSOP	• Sample size: *n* = 22 • Age (mean years): 86 • Female %: 73 • Conditions: Mean value: Cumulative Illness Rating Scale: 4; MMSE score: 25; Barthel index: 81; Tinetti scale score: 18.	Physical exercise rehabilitation with nutritional intervention	—	• Muscle mass: Skeletal muscle mass index (+) • Muscle strength: Hand grip strength (+) • Physical performance: Gait speed (+), Short Physical Performance Battery test score (+)	• Body weight (+), body mass index (+) • Vitamin D level (+)		
Lin et al. (2020) Taiwan	Nursing home	Skeletal Muscle Mass Index plus Handgrip Strength (with cut‐off points for Malaysia population)	• Sample size: *n* = 17 • Age (average, years): 82 • Female %: 29 • Conditions: 24% with diabetes, >50% experienced fall(s) and in the prefrail stage.	Whole‐body vibration training	—	• Muscle strength: Hand grip strength (+) • Physical performance: 8‐foot up and go test time (−), 5 sit‐to‐stand test time (−), shoulder–arm flexibility (+)	• Activities of daily living (Barthel index): Highly dependent: 21–60 points (+); moderately dependent: 61–90 points (−); slightly dependent: 91–99 points (=); completely independent: 100 points (=) • Functional balance: 1‐foot balance (+)	• Sleep quality: Pittsburgh sleep quality (+)	

*Note*: (+) represents the increased direction; (−) represents the decreased direction; and (=) represents no change. Abbreviations: AWGS, the Asian Working Group for Sarcopenia; CG, control group; FNIH, the Foundation for the National Institutes of Health; IG, intervention group; SARC‐F questionnaire, Strength, Assistance walking, Rising from a chair, Climbing stairs, and Falls questionnaire.

### Risk of bias assessment

**Figure 2:**
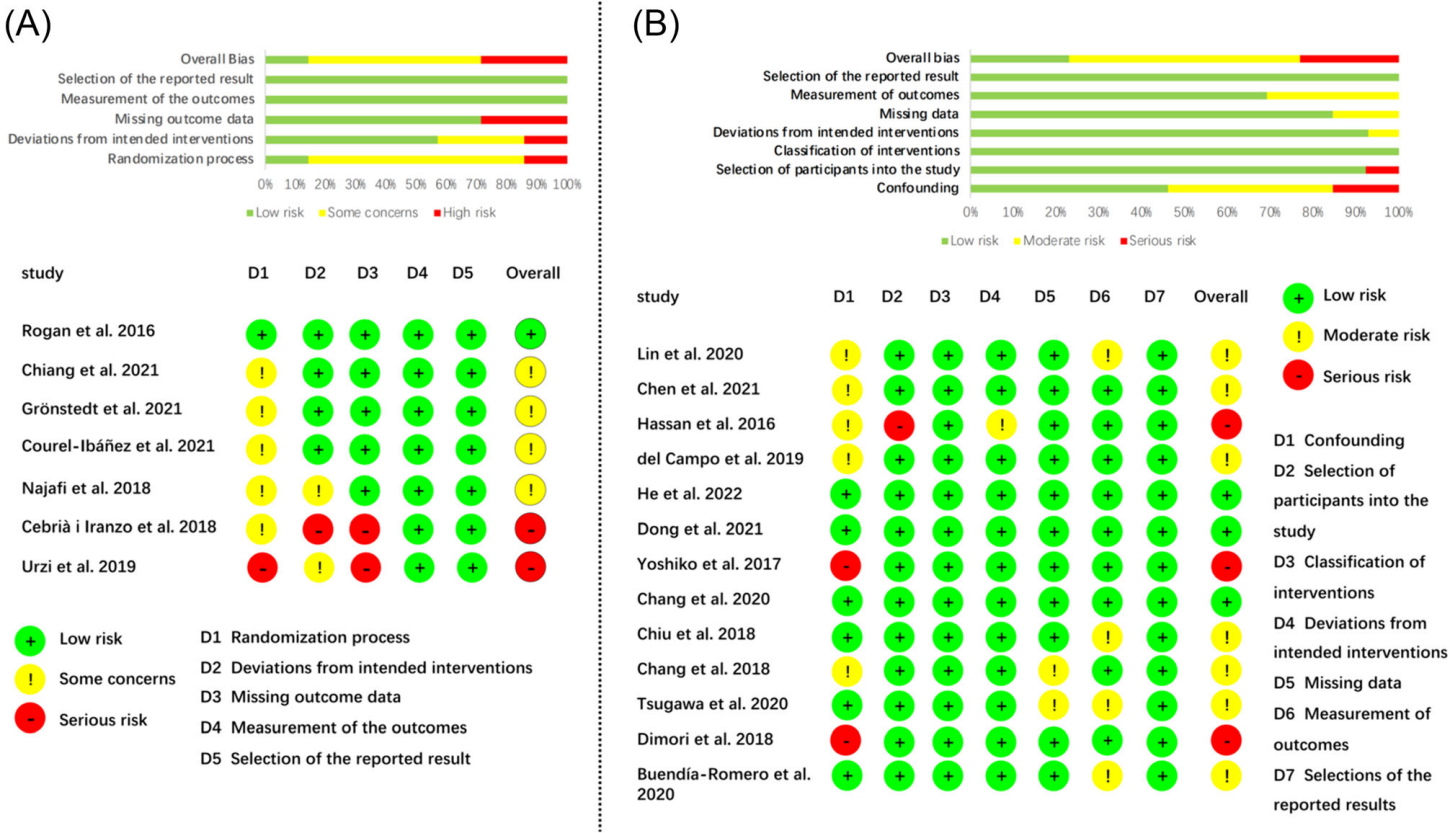
Results of the risk of bias assessment using RoB2 and ROBINS‐I. (A) Bar chart overview and per‐study risk of bias rating for RCT studies. (B) Bar chart overview and per‐study risk of bias rating for non‐RCT studies.

Version 2.0 of the Cochrane Risk of Bias tool for randomized trials (RoB)^38^ was used to evaluate the risk of bias of seven RCTs^46^, ^49^, ^50^, ^52^, ^56^, ^57^, ^58^ (*Figure* *2* *A*) and one cluster RCT.^53^ Overall, two studies were at low risk,^53^, ^57^ two studies were at serious risk^49^, ^52^ and the remaining studies (*n* = 4) had some concerns.^46^, ^50^, ^56^, ^58^ The randomization process could be a major source of risk of bias. Six of the studies did not offer information on their randomization process.^46^, ^49^, ^50^, ^52^, ^56^, ^58^ In terms of the deviations from intended interventions, none of the RCTs blinded both participants and investigators. Two studies^49^, ^52^ were rated with high risk in the missing outcome data domain because the data for outcomes were only available for approximately half of their participants. All the RCTs were at low risk in measurements of outcomes and selection of the reported result, as the measure methods were appropriate and objective in all studies, and the data and outcomes are fully documented without any bias in selection.

The Cochrane ROBINS‐I was adopted to assess the risk of bias of seven two‐arm quasi‐experimental trials^25^, ^26^, ^27^, ^45^, ^48^, ^51^, ^55^ and six single‐arm pretest–posttest design trials.^43^, ^44^, ^47^, ^54^, ^59^, ^60^ The results were shown in *Figure* *2* *B*. Overall, three studies^26^, ^45^, ^51^ were at low risk, nine studies^25^, ^26^, ^44^, ^45^, ^47^, ^48^, ^54^, ^59^, ^60^ were at moderate risk and three studies^27^, ^43^, ^55^ were at serious risk. The main source of risk of bias is confounding. Five studies that failed to report or control for important baseline characteristics (i.e., age, sex and medical conditions) were considered at moderate risk,^44^, ^47^, ^55^, ^59^, ^60^ and two studies^27^, ^43^ with notable differences between two groups of participants were rated as serious risk. The secondary source of bias is the measurement of outcomes. Four studies^25^, ^47^, ^48^, ^54^ had a moderate risk in terms of this domain as they used assessor‐subjective outcomes and did not describe blinding methods of assessment. All studies were at low risk of bias for the classification of interventions and the selection of the reported results.

### Characteristics of the included interventions

Several types of interventions targeting sedentary behaviour and physical inactivity for sarcopenia in LTCFs were identified, including resistance exercise alone (*n* = 9)^43^, ^44^, ^45^, ^49^, ^50^, ^51^, ^52^, ^55^, ^60^; multicomponent exercise, which is resistance training combined with aerobic exercise, balance training and/or endurance training (*n* = 6)^25^, ^26^, ^27^, ^54^, ^56^, ^58^; whole‐body vibration training (*n* = 3)^47^, ^57^, ^59^; aerobic exercise combined with traditional Chinese medicine exercise (*n* = 1)^53^ or cognitive training elements (*n* = 1)^48^; and sit‐to‐stand training (*n* = 1).^46^ Over half of the included studies performed the intervention three times a week for 12 weeks. Two studies performing tailored multicomponent exercise with intervention provided 5 days per week,^54^, ^56^ and one study performing sit‐to‐stand intervention provided 7 days per week.^46^ Among the included studies, the shortest duration of exercise was 4 weeks (*n* = 1),^56^ and the longest was 48 weeks (*n* = 2).^27^, ^43^ The reporting of intervention adherence was insufficient, with only 11 out of 21 included studies^25^, ^26^, ^43^, ^44^, ^46^, ^49^, ^51^, ^52^, ^54^, ^55^, ^57^ providing adherence reports. Adherence overall and by intervention type was not possible to discern due to inconsistent criteria for high adherence across these studies. The maximum level of adherence observed was 100% in one study with an intervention group (*n* = 16) and a sham group (*n* = 14).^57^ The minimum level of adherence reported was <30% of the participants completing over 75% of the whole intervention^55^ (*Table* *2*).

**Table 2:** Description of the included interventions targeting sedentary behaviour and physical inactivity

Interventions	Provider	Resources	Intervention activities	Training parameters	Tailoring	Intervention group adherence
Resistance exercise alone						
Chen et al. (2021) Progressive resistance training via VR	A well‐trained physiotherapist	• Intervention equipment and motivation: Virtual reality equipment and game software	• With VR games, conducting resistance training on the upper extremity, which includes elbow flexors and extensors; extensor carpi radialis brevis and longus. • Each VR game for 6 min, 1–2 min interval between each game.	• 2 sessions per week, ≥48 h intervals • 12 weeks • 40 min per session, 5 min warm‐up, 30 min training, 5 min cool‐down	• Selecting games based on the body stability and stationary conditions of the participants. • Increasing the weight of mass based on individuals' ability.	Not stated.
Chiang et al. (2021) Low resistance exercise training combined with milk or soymilk	Not stated	• Accessories: Sandbags, elastic bands • Intervention aids: Chair • Nutrition supplement: Milk, soymilk	• Exercise training: Chair exercise, resistance exercise with sandbags and elastic bands, balance and gait training. • Drinking 200 mL of milk or soymilk twice per day.	• 3 sessions per week • 12 weeks • 30 min per session	Not stated.	Not stated.
del Campo et al. (2019) Resistance exercise training	Not stated	• Guideline: ACSM • Accessories: Dumbbells, elastic bands	• Developing the intervention based on the ACSM guideline. • Training 2–3 sets at a moderate or high intensity using dumbbells and elastic bands. 8–12 repetitions for the 1st and 2nd months, 15 repetitions for the 3rd month. 1–3 min of rest between sets.	• 3 sessions per week • 12 weeks	• Increasing the strength and the load based on the progression of participants.	80.55% participants completed >70% intervention and 36% completed the whole.
Dimori et al. (2018) Physical exercise rehabilitation programme and nutritional interventions	Not stated	• Nutritional supplement: Vitamin D, calcium and leucine‐enriched whey protein‐based oral nutritional supplement	• Aerobic training and strength training. • Providing vitamin D, calcium and leucine‐enriched whey protein‐based oral nutritional supplement twice daily for the first 6 months and the last 3 months.	• 3 sessions per week • 48 weeks • 40 min per session, 5 min warm‐up, 30 min training, 5 min cool‐down	Not stated.	>85% participants completed >80% of the intervention.
Dong et al. (2021) Chair‐based resistance training with elastic belt	Trained researchers	• Guideline: ACSM • Education: Booklets • Intervention aids: Chair • Monitoring and tailoring: Electronic sphygmomanometer, Borg Rating of Perceived Exertion scale	• Developing the intervention based on the ACSM guideline. • Training researchers and participants with booklets prior to the intervention. • 9 chair‐based resistance actions with elastic belts. Repeating each action 12–15 times for three sets, 2–3 min of breaks between actions. • Monitoring heart rate, blood pressure and any symptoms before and during each session.	• 3 sessions per week • 12 weeks • 40–50 min per session, with 5 min warm‐up, 30–40 min training and 5 min relaxation	• Starting with the least elastic band, the intensity was adjusted according to the Borg Rating of Perceived Exertion scale and the individual's physical ability.	84% participants completed 91.6% total sessions.
Hassan et al. (2016) Progressive resistance and balance training	Trained allied health professional	• Education: Face‐to‐face training • Intervention equipment: Air‐pneumatic equipment	• Instructing providers to operate the exercise equipment and conduct the training prior to the intervention. • Using air‐pneumatic equipment to do lower and upper body, and the trunk exercises. • Balance exercises include heel and toe raise, single‐leg standing, static balance, heel to toe walking.	• 2 sessions per week • 24 weeks • 60 min per session	With Borg scale, increasing the load if comfortably completed 3 sets of 10 repetitions or increasing repetitions with the same load to 3 sets of 15 repetitions.	28.4% participants completed >75% of the whole intervention.
He et al. (2022) Resistance exercise training with elastic belt	Trained researchers	• Guideline: ACSM • Education: Face‐to‐face training • Accessories: Elastic belts • Monitoring and tailoring: Borg Rating of Perceived Exertion scale	• Developing the intervention based on the ACSM guideline. • Training researchers and participants with prior to the intervention. • Several resistance exercises actions with elastic belts. Every action maintains 5 s, 2–3 times a set and 1–2 min of breaks between sets. • Monitoring any adverse activities during the training.	• 2–3 sessions per week • 12 weeks • 40–50 min per session, with 5 min warm‐up, 30–40 min training and 5 min relaxation	• Starting with the least elastic band, the intensity was adjusted according to the Borg Rating of Perceived Exertion scale and the individual's situation.	Not stated.
Cebrià i Iranzo et al. (2018) Resistance training of peripheral muscles	Two physiotherapists not involved in assessments	• Accessories: Dumbbells, ankle/wrist weights • Motivation: Training journals • Monitoring and tailoring: Pulse oximeter, Borg Rating of Perceived Exertion scale	• 10 isotonic resistance exercises with dumbbells and ankle/wrist weights, 12 repetitions for each one. • Monitoring SpO_2_ and heart frequency during training.	• 3 sessions per week • 12 weeks • 30–40 min per session, with 5‐min warm‐up, 20–30 min resistance exercise and 5‐min cool‐down	• Resistance exercises were performed with a workload adjusted to 40–60% of maximal isometric muscles strength. • Recording the increases in training workload and their perceived exertional efforts (Borg scale).	40.7% participants completed ≥80% of the intervention.
Urzi et al. (2019) Progressive elastic resistance training of moderate intensity	Not stated	• Accessories: Elastic bands • Intervention aids: Chair • Monitoring and tailoring: Borg Rating of Perceived Exertion scale	• Elastic resistance training includes resistance chair squats; band seated: biceps curl, seated row, knee extension, leg press and hip abduction, knee flexion and calf rise. • Maintaining the exercise intensity on the Borg Rating of Perceived Exertion scale level ‘somewhat hard’.	• 3 sessions per week • 12 weeks • 45–50 min per session	• Borg Rating of Perceived Exertion scale level was used to identify the moderate intensity of the intervention for each participant.	Average adherence to intervention: 87.6% (±8.8%).
Multicomponent exercise (resistance training combined with aerobic exercise, balance training and/or endurance training)						
Buendía‐Romero et al. (2020) Courel‐Ibáñez et al. (2022) Vivifrail tailored, multicomponent exercise programme	Multidiscipline health team	• Education: Mobile application, booklets • Accessories: Bottles of water (used as dumbbells), grip balls • Intervention aids: Chair, walking/standing aids	• Dividing participants into A, B, C, D groups (ranging from disability to robust) by individual assessment on functional ability. • Introducing the training participants with booklets and mobile application. One familiarization week prior to intervention. • Multicomponent exercise: Resistance, balance, flexibility and cardiovascular endurance exercises. • Monitoring safety and tolerance, filling the intervention diary and recording participants' feelings.	• 5 sessions per week • 24 and 4 weeks • 30–45 min per session in A group, 45–60 min per session in B, C, D groups	• The individualized functional ability assessment to recruit participants into corresponding types. • In each type, loads of the exercise will be adjusted according to participants' physical ability and tolerance.	78.6% participants completed 96% of the intervention in Buendía‐Romero et al.'s study. Not stated in Courel‐Ibáñez et al.'s study.
Chang et al. (2020) Chiu et al. (2018) Chair‐based resistance training	A professional health trainer	• Accessories: Grip ball, sandbags • Intervention aids: Chair • Motivation: Nostalgic music, games • Monitoring and tailoring: Borg Rating of Perceived Exertion scale	• Developing the intervention by consulting with a multidisciplinary health care team. • Introducing the interventions and assessment tool to participants with pictures. Familiarization of the motions of training without any load. • Training focusing on upper body and lower extremities. Chair muscle strength training by using a grip ball or sandbags on wrist or ankle joints, along with nostalgic music and games. 3 sets of 4–10 repetitions with 30‐s rest for each session.	• 2 sessions per week, ≥48 h intervals • 12 weeks • 60 min per session, with warm‐up, training and cool‐down parts	• Checking health conditions and grip strength before giving load. • Adjusting the load based on feedback and Borg scale score.	66.7% completed >50% of the whole interventions in both Chang et al.'s and Chiu et al.'s studies.
Najafi et al. (2018) Fun and regular physical activities	A certified clinical exercise specialist and a general practitioner	• Motivation: Plastic balls, catch‐a‐colour rockets, wands, Audubon bird, stretch bands • Intervention aids: Walking aids	• Strength, balance, endurance and walking activities with plastic balls, catch‐a‐colour rockets, wands, Audubon bird and stretch bands. • Daily walking for half an hour and stretching.	• 3 sessions per week • 8 weeks • 20 min per session	Not stated.	Not stated.
Yoshiko et al. (2017) Resistance and endurance training	A physical therapist	• Education: Face‐to‐face training • Intervention equipment: Hydraulic‐resistance machine, recumbent stepper • Accessories: Resistance tube and ball • Intervention aids: Chair • Monitoring and tailoring: Borg Rating of Perceived Exertion scale	• Instructing participants of the motion for each exercise. • Resistance training using hydraulic‐resistance machine, resistance tube and ball. Endurance training using recumbent stepper.	• 1–2 sessions per week • 48 weeks • 150 min per session, with 20 min warm‐up, 40–45 min training and 20 min cool‐down	Starting with the least load, adjusting the intensity to 11–12 points of Borg scale and 40% of maximum effort.	Not stated.
Whole‐body vibration training						
Chang et al. (2018) Lin et al. (2020) Whole‐body vibration intervention	Researchers	• Intervention equipment: Whole‐body vibration machine (i‐vib6050 model)	• Standing on a vibration and stimulation‐generating platform with a vibration frequency and amplitude of 12 Hz and 3 mm. • Monitoring safety during the intervention.	• 3 sessions per week • 12 weeks • 10 repetitions per session	Not stated.	Not stated.
Rogan et al. (2016) Whole‐body vibration (WBV) and a dance video game (DVG)	A professional exercise instructor	• Whole‐body vibration platform • Intervention aids: Chair • Motivation: Dance video game, motivation–volition strategies	• Familiarization 1 week prior to intervention and task tutorial. • WBV: Parallel standing with slight flexion of hip, knee and ankle joints. Each session, five 1‐min vibration periods with 1‐min seated break between sets. • Dance video game with motivation–volition strategies each session.	• 3 sessions per week • 8 weeks • WBV: 3–6 Hz, amplitude 4 mm • DVG: 4× 2–3 min songs, 30‐s break after each song	• WBV: Frequency increased depending on capabilities and feedback. Parallel standing changed to tandem standing and dynamic squat movements. •DVG: Beats per minute and difficulty level adapted based on performance.	100% adherence.
Others						
Grönstedt et al. (2020) Sit‐to‐stand exercises combined with oral nutritional supplement	Nursing home staff	• Education: Face‐to‐face training class • Motivation: Designed intervention diary • Intervention aids: Walking/standing aids • Oral nutritional supplement	• Informing and training staff before intervention. • Encouraging and supporting participants to get up from a chair to a standing position frequently in daily activities. • Nutrition supplement: 125 mL, 18 g of protein, 300 kcal; Fortimel Compact Protein. • Recording the occasions of sit‐to‐stand and consumed nutrition supplement.	• 4 occasions everyday • 12 weeks • Nutrition supplement twice daily for 7 days per week	Not stated.	44% with high adherence to the sit‐to‐stand intervention.
Tsugawa et al. (2020) Exercise with gymnastics and balls recreation	A physiotherapist and a clinical physician	• Education: Face‐to‐face training • Accessories: Balls, sticks • Intervention aids: Standing aids	• Developing the intervention based on previous studies and residents' condition. • Training intervention providers. • Training of 15 min of gymnastics, 10 min of recreation with balls and standing up training for 5 min. • Monitoring safety and managing risk for accidents and other adverse events during the class.	• 2 sessions per week • 48 weeks • 40 min per session, 5 min warm‐up, 30 min training	Not stated.	Not stated.
Tung et al. (2022) Chair‐based Vitality Acupunch exercise	Two instructors, who are staff in the institution.	• Education: Face‐to‐face teaching • Intervention aids: Chair	• Modifying the intervention based on the Healthy Beat Acupunch exercise programme. • Instructing participants to stimulate the 14 meridians with a hollow fist. • Five fist styles were applied in a seated position. The three phases are activating qi and blood, punching meridians and relaxing the body and mind.	• 3 sessions per week • 24 weeks • 45–50 min per session	Not stated.	Not stated.

Abbreviations: ACSM, the American College of Sports Medicine; DVG, dance video game; VR, virtual reality; WBV, whole‐body vibration.

### Developed Theory of Change logic model

**Figure 3:**
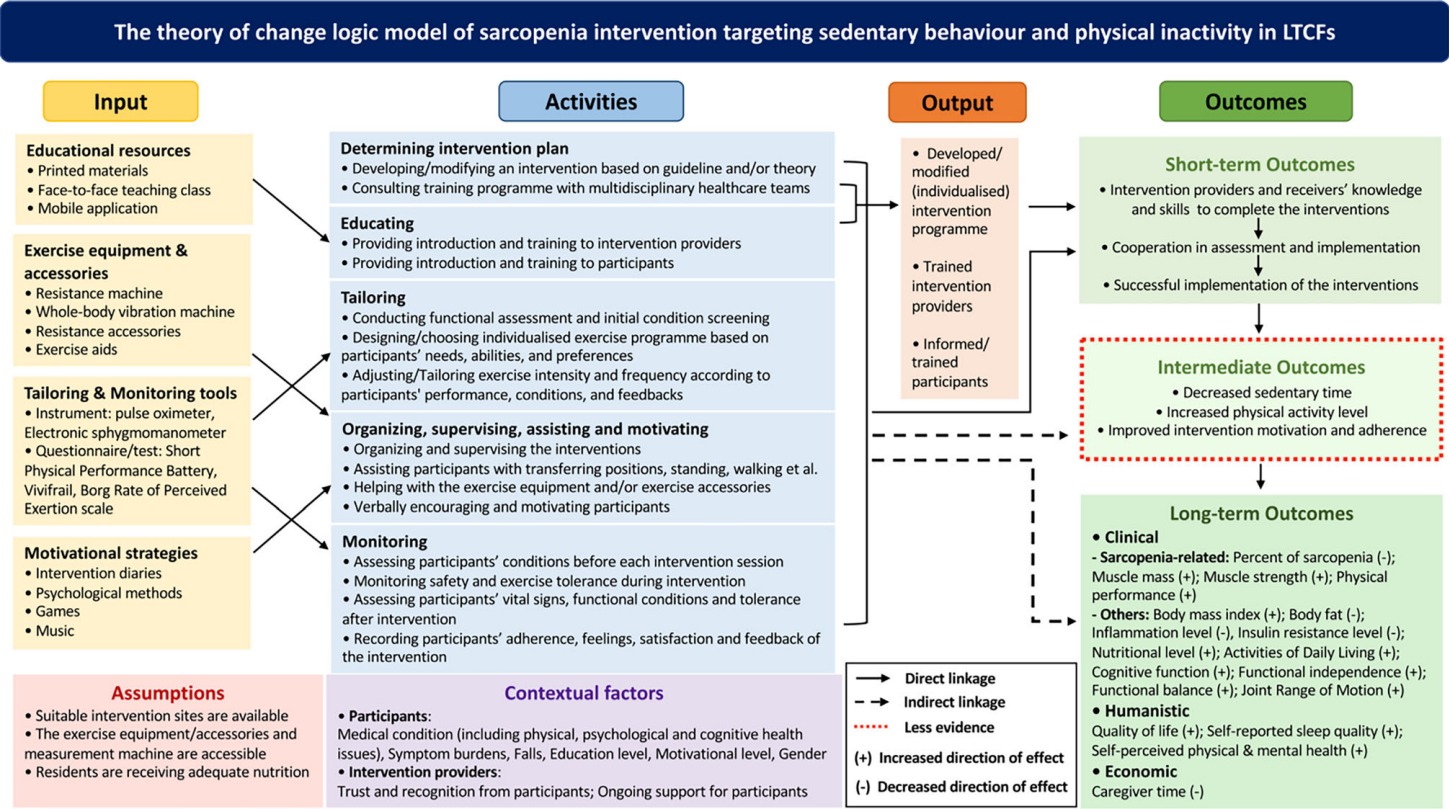
Developed Theory of Change logic model of sarcopenia intervention targeting sedentary behaviour and physical inactivity in long‐term care facilities.

*Figure* *3* illustrates a Theory of Change logic model for sarcopenia intervention targeting sedentary behaviour and physical inactivity in LTCFs. Derived from the outcomes in *Table* *1* and the descriptions in *Table* *2*, this logic model provides a condensed overview of the included interventions. It outlines the resources, processes and events expected from researchers and LTCFs to conduct sarcopenia interventions. Inputs such as educational resources, exercise equipment, tailoring and monitoring tools and motivational strategies are required for intervention activities like educating, tailoring, monitoring and motivating. These activities yield outputs such as developed intervention programmes, trained providers and informed participants, fostering increased knowledge, skills, cooperation and successful implementation (short‐term outcomes). These activities and outcomes may influence participants' motivation, adherence, sedentary time and physical activity levels (intermediate outcomes), subsequently impacting clinical indicators, humanistic status and economic investments (long‐term outcomes).

### Inputs of the included interventions

Based on the studies synthesized, four primary categories of inputs were identified: (1) educational resources; (2) exercise equipment and accessories; (3) tailoring and monitoring tools; and (4) motivational strategies. Educational resources included both printed learning materials (e.g., booklets and pictures)^25^, ^26^, ^51^, ^54^, ^56^ and face‐to‐face teaching.^27^, ^48^, ^53^, ^57^ Two studies also used a specially designed mobile application (Vivifrail) to support their interventions.^54^, ^56^ As to the category of exercise equipment and accessories, resistance exercise equipment (e.g., hydraulic‐resistance machine)^27^, ^55^ and resistance exercise accessories (e.g., elastic bands and dumbbells)^25^, ^26^, ^44^, ^45^, ^49^, ^50^, ^51^, ^52^, ^54^, ^56^ are the most common, followed by whole‐body vibration machines.^47^, ^57^, ^59^ Additionally, chairs, standing aids and walking aids were important accessories to support participants during the interventions.^25^, ^26^, ^44^, ^45^, ^49^, ^50^, ^51^, ^52^, ^54^, ^56^ Regarding tools for tailoring and monitoring, instruments like a pulse oximeter and an electronic sphygmomanometer measuring participants' vital signs were used to assess condition before each intervention session and monitor safety during interventions.^51^, ^52^ Questionnaires like the Borg Rating of Perceived Exertion scale measuring intervention tolerance^25^, ^26^, ^27^, ^45^, ^49^, ^51^, ^52^ and tests like the Vivifrail and Short Physical Performance Battery^54^, ^56^ were applied to monitor and tailor the intervention for participants. Motivational strategies were diverse. Intervention diaries and adherence charts were used to encourage intervention engagement.^54^, ^56^ Different types of games were used to stimulate interests and motivate participants, such as interactive group games (e.g., with plastic balls),^58^ virtual reality games^60^ and dance video games.^57^ Music^25^, ^26^ and psychological strategies (e.g., motivation–volition strategy)^57^ were also used to increase participation and adherence (*Table* *2* and *Figure* *3*).

### Activities of the included interventions

The activities in the interventions were grouped into five categories: determining the intervention plan; educating; tailoring; organizing, supervising, assisting and motivating; and monitoring. For determining the intervention plan, some studies developed or adapted interventions based on exercise theory, guidelines and existing programmes,^25^, ^26^, ^44^, ^45^, ^48^, ^51^, ^53^, ^60^ often consulting multidisciplinary health care teams.^25^, ^26^ Educating involved providing instruction and training to both intervention providers^45^, ^46^, ^48^, ^51^, ^53^, ^55^ and participants.^27^, ^45^, ^48^, ^51^, ^54^, ^56^, ^57^ Tailoring included conducting functional assessments and screenings to customize exercise interventions,^25^, ^26^, ^27^, ^54^, ^56^ with adjustments made based on assessment results and participant feedback.^25^, ^26^, ^27^, ^44^, ^45^, ^49^, ^51^, ^52^, ^54^, ^55^, ^56^, ^57^, ^60^ Organizing, supervising, assisting and motivating were key responsibilities of intervention providers, including organizing and overseeing face‐to‐face interventions,^25^, ^26^, ^27^, ^43^, ^44^, ^45^, ^46^, ^47^, ^48^, ^49^, ^50^, ^51^, ^52^, ^53^, ^54^, ^55^, ^56^, ^57^, ^58^, ^59^, ^60^ assisting with mobility,^46^, ^47^, ^48^, ^54^, ^56^, ^57^, ^58^, ^59^ operating exercise equipment^25^, ^26^, ^27^, ^44^, ^45^, ^47^, ^48^, ^49^, ^51^, ^52^, ^54^, ^55^, ^56^, ^57^, ^58^, ^60^ and providing verbal encouragement.^27^, ^57^ Monitoring involved evaluating participants' vital signs and symptoms before each session to ensure they were prepared for the intervention.^51^, ^52^ Safety and exercise tolerance were monitored during sessions, with vital signs and adverse events recorded.^45^, ^47^, ^48^, ^51^, ^52^, ^54^, ^56^, ^59^ After sessions, vital signs, functional conditions and training tolerance were reassessed,^25^, ^26^, ^27^, ^45^, ^49^, ^51^, ^54^, ^55^, ^56^, ^57^ and participants' adherence, experience, satisfaction and feedback were recorded^25^, ^26^, ^27^, ^46^, ^54^, ^56^ (*Table* *2* and *Figure* *3*).

### Outputs and outcomes of the included interventions

Based on the description of the interventions, three outputs of the included interventions were identified: developed/modified intervention,^25^, ^26^, ^27^, ^44^, ^45^, ^46^, ^48^, ^51^, ^53^, ^54^, ^56^, ^57^, ^60^ trained intervention providers^45^, ^46^, ^51^, ^53^, ^54^, ^55^, ^56^ and informed/trained participants.^27^, ^45^, ^46^, ^48^, ^51^, ^53^, ^54^, ^56^, ^57^

Identified short‐term outcomes mainly concerned providing educational materials and training to intervention providers and residents to ensure their understanding of the interventions and equip them with the skills needed for the intervention.^25^, ^26^, ^27^, ^45^, ^46^, ^48^, ^51^, ^53^, ^55^, ^57^ All the included studies completed their planned interventions with providers and residents' cooperation in assessment and implementation. Overall, short‐term outcomes comprised increased knowledge and skills of the interventions among LTCF staff and residents, cooperation in assessment and implementation and successful implementation of the interventions. We hypothesize a sequential relationship wherein enhanced knowledge and skills are expected to precede effective cooperation, ultimately leading to successful implementation.

All the included interventions were exercise‐based interventions, which have a direct impact on sedentary time and physical activity levels. Some studies also used motivational enhancement methods to ensure good adherence.^25^, ^26^, ^54^, ^56^, ^57^, ^58^, ^60^ Therefore, sedentary time, physical activity levels and adherence were regarded as intermediate outcomes. Adherence to the intervention has been reported in some studies,^25^, ^26^, ^43^, ^44^, ^46^, ^49^, ^51^, ^52^, ^54^, ^55^, ^57^ while sedentary time and levels of physical activity were not measured in any included studies (*Figure* *3* and *Table* *1*).

The studies reported long‐term outcomes comprising ECHO. The economic outcome is confined to one study reporting caregiver time as one of the outcomes.^46^ As to clinical outcomes, three studies measured changes in the percentage of sarcopenia. Among them, two studies^48^, ^50^ utilized AWGS criteria for diagnosing sarcopenia, while one study^27^ used EWGSOP criteria. Additionally, changes in all three sarcopenia parameters (muscle mass, muscle strength and physical performance) were measured in 11 studies.^27^, ^43^, ^44^, ^45^, ^49^, ^50^, ^51^, ^52^, ^55^, ^59^, ^60^ Some studies focused solely on changes in one or two sarcopenia parameters. Specifically, four studies^47^, ^54^, ^56^, ^58^ measured muscle strength and physical performance; two studies^25^, ^48^ measured muscle mass and muscle strength; and two other studies^55^, ^57^ only assessed physical performance. Other outcomes measured included anthropometric outcomes (e.g., body mass index),^27^, ^43^, ^44^, ^45^, ^46^, ^49^, ^50^, ^55^, ^60^ clinical laboratory measures outcomes (e.g., inflammation level)^27^, ^43^, ^46^, ^49^, ^50^ and clinician‐reported outcomes (e.g., activities of daily living).^25^, ^46^, ^47^, ^48^, ^53^, ^54^ The most frequently measured humanistic outcome (i.e., patient‐oriented outcome)^61^ was the quality of life,^26^, ^45^, ^46^, ^59^ followed by self‐perceived physical and mental health^26^, ^48^, ^54^ and self‐reported sleep quality.^47^ The specific indicators for each outcome have been shown in *Table* *1* and *Figure* *3*.

### Assumptions and contextual factors

We initially identified three assumptions for the interventions based on the information from the included studies: Suitable intervention sites are available; the exercise equipment and/or accessories and measurement machine are accessible; and residents are receiving adequate nutrition. From the included studies, factors affecting intervention adherence were identified by participants (LTCF residents) and intervention providers and recognized as contextual factors. First, the medical conditions (i.e., physical, psychological and cognitive health issues),^25^, ^26^, ^27^, ^45^, ^46^, ^51^, ^52^, ^55^, ^58^ symptom burdens (physical pain and fatigue due to assessment)^27^, ^59^ and falls^44^, ^52^ are the main reasons for participants to withdraw from interventions. Education level and motivational level also affected the adherence to interventions.^25^, ^27^ One study reported variation in exercise preferences by gender.^25^ Second, intervention providers that are trusted and recognized by participants and provide ongoing support to participants could significantly contribute to the adherence of interventions.^55^, ^58^

## Discussion

This systematic review included 21 interventional studies targeting sedentary behaviour and physical inactivity in LTCF residents at risk of or with sarcopenia. Most studies focused on resistance training and used sarcopenia‐related measures, such as muscle mass, strength and physical performance, as primary outcomes. However, intervention development was poorly reported, and adherence reporting was insufficient. Strategies to improve implementation included education and training, tailoring, monitoring and motivating. Intermediate outcomes, such as sedentary time and physical activity levels, were rarely considered. A novel logic model was developed to synthesize the interventions' inputs, activities, outputs, outcomes, assumptions and contextual factors (*Figure* *3*). This model aids in developing complex interventions for sarcopenia by outlining specific components, desired outcomes, causal relationships and assumptions.

### Guidelines specifically for long‐term care facility residents need to be referred

The development of the included interventions was generally poorly reported, with only two studies utilizing the well‐developed Vivifrail programme.^54^, ^56^ Seven studies briefly described their intervention development, often based on previous interventions^25^, ^26^, ^48^, ^53^ or the American College of Sports Medicine (ACSM) guidelines.^44^, ^45^, ^51^ Notably, Vivifrail was initially designed for community‐dwelling and hospital‐based older adults, not LTCFs. The ACSM guidelines also focus on the primary prevention of noncommunicable diseases in healthy community‐dwelling older adults.^62^, ^63^ Given the specific needs of LTCF residents, including functional limitations and multimorbidity, these guidelines may not be the most suitable for this population. The exercise goals for LTCF residents should prioritize maintaining functional capacity and improving quality of life.^64^ Unsuitable guidelines applied may possibly contribute to low adherence. Exercise guidelines for LTCF residents were published in 2016 and updated in 2023.^64^, ^65^ Despite being available in 90% of the included studies, none explicitly referenced these guidelines. The LTCF guidelines highlight reducing sedentary behaviour for all residents and personalizing physical activity programmes based on individual needs and levels of dependence. These guidelines are likely more feasible and effective for LTCF residents at risk of or with sarcopenia.

### Sedentary behaviour deserves more consideration

The first level of the LTCF physical activity guidelines, which seeks to diminish sedentary behaviour among all residents, places significant emphasis on the reduction of sedentary time.^64^, ^65^ Limited use of the LTCF physical activity guidelines in the included studies meant insufficient consideration is given to sedentary behaviour for LTCF residents at risk of or with sarcopenia. Only one included study specifically addressed sedentary behaviour,^46^ and only five of the remaining 20 included studies^43^, ^44^, ^49^, ^51^, ^53^ incorporated the reduction of sedentary time as an intervention component. The methods used to decrease sedentary time included training to transition from sitting to standing, standing training or walking practice. Despite attempts, few of the interventions implemented met the criteria in the LTCF guidelines,^64^, ^65^ which advise interrupting the sedentary time of LTCF residents with short breaks lasting 2–5 min, 2 or 3 times daily. Indeed, breaking up prolonged periods of sitting with different types and levels of physical activity can be beneficial.^64^, ^65^ The study embedding sit‐to‐stand exercises into daily life offers a fresh perspective. By encouraging and supporting residents to move from sitting to standing frequently in their daily activities, residents' position transfer performance improved with good adherence.^46^

### Enhancing adherence to interventions is imperative

The insufficient reporting of intervention adherence, combined with inconsistent criteria for high adherence across studies, makes it difficult to accurately discern adherence overall and by intervention type. However, adherence is a key factor in interpreting the results of exercise interventions^66^ to validate interventions' success, improve programme design and ensure optimal health outcomes for participants. Therefore, it is crucial to pursue innovative methods to monitor adherence. A recommended approach is the implementation of a scoring system to measure and report adherence. This provides a way to set intervention goals and track participant progress, providing an overall assessment of adherence to the programme and/or its components.^66^ This method has demonstrated success in monitoring adherence to exercise‐based interventions.^66^, ^67^

Moreover, low adherence can significantly undermine the effectiveness of interventions, potentially skewing the assessment of intervention impact and leading to unreliable interpretations of the results.^66^ Thus, it is imperative to explore effective ways to enhance robust adherence to interventions targeting sedentary behaviour and physical inactivity in LTCFs. Main factors influencing adherence, including lack of knowledge, risk of adverse events, poor exercise tolerance due to medical condition or symptom burdens, lack of motivation and loss of interest in intervention, have been identified in the included studies.^25^, ^27^, ^45^, ^46^, ^51^, ^55^, ^58^, ^59^ Drawing from the major risk factors and evidence‐based methods, we identified four areas to enhance adherence: (1) education, (2) monitoring, (3) personalization (tailoring) and (4) motivation and interest promotion:

1. Educating participants and intervention providers before and during conducting interventions is essential. Nearly half of the included studies did this. Knowledge, attitude, belief and practice mode (KABP) hold that knowledge and information are the basis for establishing positive and correct beliefs and attitudes, thus promoting health‐related behaviours.^68^ This emphasizes the importance of providing ongoing education to participants. Besides, ageism is the stereotyping, prejudice and discrimination against people based on their age.^69^ It has been shown that ageism against older adults negatively affects their health, well‐being, behaviour and the quality of health care they receive.^70^, ^71^, ^72^ Educating intervention providers can not only allow them to provide ongoing guidance and support to participants during interventions at a good standard but also serve as a potential solution to mitigate ageism.^73^
2. Monitoring safety and exercise tolerance was conducted in several included studies. Generally, the main purpose of the monitoring process is to ensure that participants are able to adapt physiologically to the training stimulus and that the training load is appropriate for the individual.^74^ Another role of monitoring is to ensure sufficient recovery between training sessions and periods.^75^ However, for older adults, safety monitoring to prevent accidents during interventions (e.g., falls) and to timely react to any undesirable response can be priorities.^75^ Both subjective measures (e.g., Borg Rating of Perceived Exertion scale) and objective measures (e.g., vital signs) were used in the included studies.^25^, ^26^, ^27^, ^45^, ^49^, ^51^, ^52^, ^55^ This is basically in line with the latest exercise guidelines for residents living in LTCFs.^65^ However, the appropriate method needs to be selected according to the different conditions of residents (e.g., cognitive impairment affecting subjective results and medication use affecting objective results).^65^ These monitoring assessments and documentation can also provide feedback to adjust intervention plans for individual participants (i.e., weekly step increments, resistance/aerobic training intensities and exercise selection). This additionally facilitates adherence to interventions.
3. Personalization (tailoring) of interventions for the individual considering diverse medical conditions, symptom burden and preferences of each LTCF resident could be ideal. The updated exercise guideline for LTCF residents emphasizes personalized physical activity programmes tailored to individual needs and levels of dependence.^65^ Two studies included in the review conducted initial assessments of physical ability and fall risk to customize exercise programmes, resulting in high adherence and effectiveness.^54^, ^56^ A scoping review also indicates that personalized interventions for improving physical performance are more effective than generic, one‐size‐fits‐all approaches.^76^ Personalization is essential, particularly for individuals with cognitive impairment. It could be achieved in different ways, such as holistic assessment considering physical, psychological, cognitive and social factors; engaging participants with ‘listening’ beyond merely ‘education’; utilizing state‐of‐the‐art equipment with real‐time biofeedback like force gauges and light sensors to customize exercises^76^; applying user‐friendly technologies such as applications to support participation^77^; and simplifying instructions and providing visual and tactile cues, as well as involving family members.^78^ Though personalized interventions may impose additional demands on facilities in terms of personnel, material resources and financial investments, they may constitute a cost‐saving strategy in the long term, as their benefits outweigh the costs.^79^
4. Motivation and interest promotion within the intervention are vital for adherence. The monotony of the intervention and the participants' low drive are common factors that impact their compliance. Incorporating recreational activities such as games and/or music, along with behaviour change theory, has been used as a strategy to address these problems. For example, video game dancing allows participants to promote fast, rhythmic and accurate foot movements and improve higher cognitive processing during interesting game play.^80^ In addition, increased physiological arousal^81^ and positive subjective experiences^82^ are the two most common ways to explain the positive effects of music in exercise. Adding music at a tempo matched with physical exercise training helps to reduce perceived exertion, lower oxygen consumption and improve exercise intensity and endurance.^83^, ^84^ Notably, one included study^57^ achieved 100% adherence. It used behaviour change theory (motivation–volition strategy)^85^ to teach participants cognitive‐behavioural strategies of goal setting, action planning, barrier management and self‐monitoring. However, the strategy embedded in its interventions was not specifically described, limiting its reproducibility. There are multiple behaviour change models and theories. However, their complexity often impedes health care professionals from applying them well in routine care.^86^ The Behaviour Change Wheel (BCW) is a comprehensive framework for designing interventions by explicitly integrating behaviour theory and target mechanisms of action within the intervention.^87^ It may be an appropriate tool to help researchers and health care professionals embed behaviour change theories into interventions targeting sedentary behaviour and physical inactivity to manage sarcopenia in LTCFs.

### An agreed set of comprehensive outcome measures is essential

The reported outcome indicators of intervention effects (long‐term outcomes) were divided into three main categories according to the concept of the ECHO Model.^42^ Both clinical (e.g., sarcopenia parameters) and humanistic outcomes (e.g., quality of life) have been well applied and reported, while economic outcomes were less considered. Caregiver time is the only reported economic outcome from the included studies. The intervention that reported economic outcomes was sit‐to‐stand exercises combined with oral nutritional supplements.^46^ Although the researchers had intended to examine health care costs, they did not do so as the use of resources was quite similar across the board.^46^ This, though, is a narrow view of economic evaluation, encompassing only formal care costs. For personalized interventions with multiple components, it is crucial to conduct further and full economic outcome assessments and analyses to consider intervention use in routine care. For clinical long‐term outcomes, despite the use of validated measures, notable variability is evident in sarcopenia diagnostic criteria across studies, directly impacting reported prevalence. For instance, using EWGSOP criteria, sarcopenia prevalence is higher compared to EWGSOP2 (23% vs. 10%, respectively).^9^ Achieving consensus on diagnostic criteria would improve comparability and aid translation into clinical practice.^9^ Hence, employing consistent parameters for sarcopenia diagnosis is advisable, despite potential variations in cut‐off points worldwide attributable to ethnic or gender differences.

Additionally, sedentary time and physical activity levels could be important intermediate outcomes. However, these were hardly assessed in the included studies. Collecting reliable physical activity levels among people living in LTCF is challenging. Self‐reported measures often fail to accurately capture actual physical activity levels,^88^ particularly in LTCF settings where lifestyle factors and cognitive impairments may compromise the reliability of these reports.^89^ Activity monitors (e.g., activPAL) have been used to track older adults' sedentary time and physical activity levels in LTCFs.^90^, ^91^ Data from the monitors have also been used to provide personalized feedback and positively impact motivation and emotions, enhancing adherence and addressing potential barriers as part of a behavioural strategy.^92^, ^93^ Although activity monitors are a more suitable and objective option, their introduction in LTCF settings remains limited. By addressing educational needs for both staff and residents and securing endorsements by health authorities (i.e., economic and policy support and partnerships with health organizations),^94^, ^95^, ^96^ LTCFs can more effectively incorporate activity monitors.

Overall, an agreed set of comprehensive outcome measures, taking long‐term outcomes (ECHO) as well as intermediate‐term outcomes (i.e., adherence, reduced sedentary time and increased physical activity levels) and short‐term outcomes (e.g., knowledge and skills of the intervention) into account, is required for interventions targeting sedentary behaviour and physical inactivity to manage sarcopenia in LTCFs.

### Strengths and limitations

The study presents a novel Theory of Change logic model to outline the components of exercise‐based interventions for managing sarcopenia in LTCFs and their connections to desired outcomes. This model delineates potential mechanisms for intervention effectiveness and identifies gaps necessary to achieve desired outcomes, aiding comprehension of interventions targeting sedentary behaviour and physical inactivity in sarcopenia management. Moreover, it can inform the development of personalized exercise‐based interventions for sarcopenia in LTCFs. The moderate risk of bias in the included studies also adds to the robustness of our findings. However, our study has limitations. We did not conduct a meta‐analysis due to the heterogeneous nature of the included studies, preventing the determination of the most effective intervention method. Additionally, the logic model was developed from included studies without stakeholder input, potentially overlooking practical considerations for developing complex interventions. Further stakeholder consultations could refine the logic model and address these limitations.

## Conclusions

A novel logic model was devised to depict the components of interventions aimed at addressing sedentary behaviour and physical inactivity to manage sarcopenia in LTCFs. It identifies areas for enhancing these interventions, including the adoption of guidelines tailored for LTCF residents, increased focus on addressing sedentary behaviour and enhancing adherence through improved education, monitoring, tailoring and motivation. Additionally, it recommends developing a standardized set of outcome measures for future sarcopenia interventions in LTCFs.

## Conflict of interest statement

The authors declare that they do not have any potential conflicts of interest to disclose.

## Supporting information


**Data S1.** Supporting Information.
