# Supplementary material for: Sarcopenia interventions in long‐term care facilities targeting sedentary behaviour and physical inactivity: A systematic review
Source: J Cachexia Sarcopenia Muscle. 2024 Sep 18;15(6):2208–33. doi: 10.1002/jcsm.13576 (PMC11634478; doi:10.1002/jcsm.13576)
Supplement: Supplementary file 1 — Data S1. Supporting Information. [file JCSM-15-2208-s001.docx]

**Number of results for each database**

| **Database** | **Number of results** |
| --- | --- |
| **MEDLINE** | **565** |
| **Embase** | **221** |
| **PsycInfo** | **305** |
| **CINAHL** | **171** |
| **Web of Science** | **101** |
| **Cochrane Library** | **121** |
| **Wanfang** | **178** |
| **CNKI** | **70** |
| **Total** | **1732** |

**from inception to 26^th^ Feb. 2024**

**Search strategy**

| **Table 1** | **Search strategy in MEDLINE database via Ovid** |
| --- | --- |
| 1 | exp Sarcopenia/ or exp Muscular atrophy/ or exp Muscle weakness/ |
| 2 | (sarcopenia* or sarcopenias* or muscle atrophy* or muscle atrophies* or muscular atrophy* or muscular atrophies* or muscle weakness* or muscular weakness*).ti,ab. |
| 3 | (muscle mass* or muscular mass*).mp. |
| 4 | exp Muscle strength/ or exp Hand strength/ |
| 5 | (muscle strength* or hand strength* or hand strengths* or grip* or grips* or grasp* or grasps*).ti,ab. |
| 6 | exp Physical functional performance/ or exp Walking speed/ |
| 7 | (functional performance* or functional performances* or physical performance* or physical performances* or physical functional performance* or physical functional  performances* or gait speed* or gait speeds* or walking pace* or walking paces* or walking speed* or walking speeds*).ti,ab. |
| 3 | 1 or 2 or 3 or 4 or 5 or 6 or 7 |
| 4 | exp Exercise/ or exp Exercise movement techniques/ or exp Exercise Therapy/ or exp Running/ or exp Yoga/ or exp Tai Ji/ or exp Qigong/ or exp Resistance training/ or exp Sedentary behaviour/ |
| 5 | (exercise* or exercise training* or acute exercise* or aerobic exercise* or isometric exercise* or physical activity* or exercise movement techniques* or pilates training* or exercise therapy* or rehabilitation exercise* or running* or yoga* or tai chi* or tai ji* or taijiquan* or taiji* or tai ji chuan* or qigong* or qi gong* or chi kung* or weight bearing exercise program* or weight bearing strengthening program* or weight lifting exercise program* or weight lifting strengthening program* or sedentary behaviour* or sedentary behavior* or sedentary time* or sedentary lifestyle* or physical inactivity*).ti,ab. |
| 6 | 4 or 5 |
| 7 | exp Aged/ |
| 8 | (aged* or elderly*).ti,ab. |
| 9 | (older adults* or older people* or older persons*).mp. |
| 10 | 7 or 8 or 9 |
| 11 | exp Long-term care / or exp Nursing homes / or exp Residential facilities/ |
| 12 | (nursing home* or residential facility* or residential facilities* or long term care*).ti,ab. |
| 13 | (long term care facility* or nursing home* or convalescence home* or extended care facility* or skilled nursing facility* or long term medical care* or long term therapy* or long term treatment* or hospice home* or institutionalization* or housing for the elderly*).mp. |
| 14 | 11 or12 or 13 |
| 15 | 3 and 6 and 10 and 14 |

| **Table 2** | **Search strategy in Embase database via Ovid** |
| --- | --- |
| 1 | exp Sarcopenia/ or exp Muscular atrophy/ |
| 2 | (sarcopenia* or sarcopenias* or muscle atrophy* or muscle atrophies* or muscular atrophy* or muscular atrophies* or muscle weakness* or muscular weakness*).ti,ab. |
| 3 | exp Muscle mass/ or exp Muscle strength/ or exp Hand strength/ or Grip strength/ or exp Hand grip/ or exp walking speed/ or exp Physical performance/ |
| 4 | (muscle mass* or muscle strength* or hand strength* or hand strengths* or grip* or grips* or grasp* or grasps*).ti,ab. |
| 5 | exp Physical functional performance/ or exp Walking speed/ |
| 6 | (functional performance* or functional performances* or physical performance* or physical performances* or physical functional performance* or physical functional  performances* or gait speed* or gait speeds* or walking pace* or walking paces* or walking speed* or walking speeds*).ti,ab. |
| 7 | 1 or 2 or 3 or 4 or 5 or 6 |
| 8 | exp Exercise/ or exp Physical inactivity/ or exp Physical activity/ or exp Sedentary lifestyle/ or exp Yoga/ or exp Tai Chi/ or exp resistance training/ |
| 9 | (exercise* or exercise training* or acute exercise* or aerobic exercise* or isometric exercise* or physical activity* or exercise movement techniques* or pilates training* or exercise therapy* or rehabilitation exercise* or running* or yoga* or tai chi* or tai ji* or taijiquan* or taiji* or tai ji chuan* or qigong* or qi gong* or chi kung* or weight bearing exercise program* or weight bearing strengthening program* or weight lifting exercise program* or weight lifting strengthening program* or sedentary behaviour* or sedentary behavior* or sedentary time* or sedentary lifestyle* or physical inactivity* or physical activity* or resistance training).ti,ab. |
| 10 | 8 or 9 |
| 11 | exp Aged/ |
| 12 | (aged* or elderly*).ti,ab. |
| 13 | (older adults* or older people* or older persons*).mp. |
| 14 | 11 or 12 or 13 |
| 15 | exp Nursing homes/ or exp Long-term care/ or exp Residential facilities/ |
| 16 | (nursing home* or residential facility* or residential facilities* or long term care*).ti,ab. |
| 17 | (nursing home* or convalescence home* or extended care facility* or long term care facility* or long term medical care* or long term therapy* or long term treatment* or hospice home* or institutionalization* or housing for the elderly*).mp. |
| 18 | 15 or16 or 17 |
| 19 | 7 and 10 and 14 and 19 |
| 20 | exp Intervention study/ or exp Experimental study/ or Exp quasi experimental study/ |
| 21 | (intervention study* or intervention trial* or interventional studies* or interventional trails* or experimental studies* or quasi-experimental study*).ti,ab. |
| 22 | 20 or 21 |
| 23 | 19 and 22 |

| **Table 3** | **Search strategy in APA PsycInfo database via Ovid** |
| --- | --- |
| 1 | exp Muscular atrophy/ or exp Muscular Disorders/ or exp Musculoskeletal Disorders/ |
| 2 | (muscular atrophy* or muscular disorders* or musculoskeletal disorders*).ti,ab. |
| 3 | (sarcopenia* or muscle weakness* or muscular mass* or skeletal muscle index*).mp. |
| 4 | exp Physical strength/ |
| 5 | (physical strength* or hand strength* or hand grip strength* or hand grasp strength*).ti,ab. |
| 6 | exp Physical activity/ or exp Walking/ or exp Gait/ |
| 7 | (physical activity* or walking speed* or gait speed* or walking pace*).ti,ab. |
| 8 | 1 or 2 or 3 or 4 or 5 or 6 or 7 |
| 9 | exp Exercise/ or exp Aerobic exercise/ or exp Intervention/ or exp Sedentary behaviour/ or exp Physical activity/or exp Yoga/or Resistance training/ or exp Sedentary behavior/ |
| 10 | (exercise* or physical exercise* or physical fitness* or movement theory* or resistance training* or exercise training* or aerobic exercise* or physical activity* or exercise movement techniques* or pilates training* or exercise therapy* or rehabilitation exercise* or running* or yoga* or tai chi* or tai ji* or taijiquan* or taiji* or tai ji chuan* or qigong* or qi gong* or chi kung* or weight bearing exercise program* or weight bearing strengthening program* or weight lifting exercise program* or weight lifting strengthening program* or sedentary behaviour* or sedentary behavior* or screen time * or physical inactivity*).ti,ab. |
| 11 | 4 or 5 |
| 12 | exp Ageing/ or exp Older adulthood/ |
| 13 | (aged* or old age* or ageing*).ti,ab. |
| 14 | (older adults* or older people* or older persons*).mp. |
| 15 | 7 or 8 or 9 |
| 16 | exp Nursing homes/ or exp Long term care/ |
| 17 | (medical homes* or residential care institutions* or treatment facilities* or residential facility* or residential facilities* or long-term care*).ti,ab. |
| 18 | (nursing home* or convalescence home* or extended care facility* or long term care facility* or long term medical care* or long term therapy* or long term treatment* or hospice home* or institutionalization* or housing for the elderly*).mp. |
| 19 | 11 or12 or 13 |
| 20 | 8 and 11 and 15 and 19 |

| **Table 4** | **CINAHL via EBSCOhost** |
| --- | --- |
| S1 | ((MH “Sarcopenia”) OR (MH “Muscular atrophy”) OR (MH “Muscle weakness”)) OR (TI (sarcopenia* or sarcopenias* or muscle atrophy* or muscle atrophies* or muscular atrophy* or muscular atrophies* or muscle weakness* or muscular weakness*) OR AB (sarcopenia* or sarcopenias* or muscle atrophy* or muscle atrophies* or muscular atrophy* or muscular atrophies* or muscle weakness* or muscular weakness*)) |
| S2 | ((MH "Exercise") OR (MH "Resistance training") OR (MH "Therapeutic Exercise") OR (MH "Life style, Sedentary") OR (MH "Physical activity") OR (MH "Screen time") ) OR (TI (Aerobic Exercises* or Muscle Strengthening* or walking* or sport* or physical fitness* or sedentary lifestyle* or physical activity*) OR AB (Aerobic Exercises* or Muscle Strengthening* or walking* or sport* or physical fitness* or sedentary lifestyle* or physical activity*)) |
| S3 | (MH "Aged") OR (TI (aged* or elderly* or older adults* or older people* or older persons*) OR AB ( aged* or elderly* or older adults* or older people* or older persons*)) OR ( (MH "Health Services for the Aged") OR (MH "Rehabilitation, Geriatric") OR (MH "Nursing homes")) OR (TI (nursing care* or rehabilitation* or nursing homes* or residential facilities) OR AB (nursing care* or rehabilitation* or nursing homes* or residential facilities)) |
| S4 | (MH "Experimental Studies") OR ( TI (experimental studies* or clinical trials* or Nonrandomized Trials* or Controlled Before-After Studies* or Pretest-Posttest Design* or Quasi-Experimental Studies*) OR AB (experimental studies* or clinical trials* or Nonrandomized Trials* or Controlled Before-After Studies* or Pretest-Posttest Design* or Quasi-Experimental Studies*) ) OR ( (intervention study* or intervention studies* or interventional study* or interventional studies* or intervention trails* or interventional trials*)) |
| S5 | S1 AND S2 AND S3 AND S4 |

| **Table 5** | **Search strategy in Web of Science (Core collection)** |
| --- | --- |
| #1 | TS=(Sarcopenia or Muscular atrophy or Muscle weakness) |
| #2 | TS=(Exercises Exercise or Resistance training or Aerobic Exercises or Tai ji or Yoga or Qigong or Physical activity or Sedentary time reduction or sedentary breaks) |
| #3 | TS=(Aged or Older adults or Older people or Older persons or Elderly) |
| #4 | TS=(Nursing home or Long-term care or Nursing care home or Long term medical care or Extended care facility or Residential facilities) |
| #5 | TS=(Experimental study or Intervention study) |
| #6 | #1 and #2 and #3 and #4 and #5 |

| **Table 6** | **Search strategy in Cochrane Library** |
| --- | --- |
| #1 | MeSH descriptor: [Sarcopenia] explode all trees |
| #2 | ((sarcopenia*) or (sarcopenias*) or (muscular atrophy*) or (muscle weakness*)) ti, ab, kw |
| #3 | #1 or #2 |
| #4 | MeSH descriptor: [Exercise] explode all trees |
| #5 | ((exercise*) or (physical exercise*) or (physical fitness*) or (movement*) or (exercise training*) or (aerobic exercises*) or (physical activity*) or (exercise therapy*) or (rehabilitation exercise*) or (running*) or (muscle strengthening*) or (sport*) or (walking*) or (physical fitness*) or (sedentary lifestyle intervention*) or (yoga*) or (Taiji*) or (Qigong*) or (taijiquan*) or (chi kung*) or (weight bearing exercise programme*) or (weight bearing strengthening*) or (resistance training*)) ti, ab, kw |
| #6 | #4 or #5 |
| #7 | MeSH descriptor: [Aged, 80 and over] explode all trees |
| #8 | ((aged*) or (elderly*) or (older adults*) or (older people*) or (older persons*)) ti, ab, kw |
| #9 | #7 or #8 |
| #10 | MeSH descriptor: [Nursing Homes] explode all trees |
| #11 | ((nursing home*) or (convalescence home*) or (extended care facility*) or (long term care facility*) or (long term medical care*) or (long term therapy*) or (long term treatment*) or (hospice home*) or (institutionalization*)) ti, ab, kw |
| #12 | #10 or #11 |
| #13 | MeSH descriptor: [Clinical Trial] explode all trees |
| #14 | ((clinical trial*) or (intervention study*) or (interventional study*) or (intervention studies*) or (interventional studies*)) ti, ab, kw |
| #15 | #13 or #14 |
| #16 | #3 and #6 and #9 and #12 and #15 |

| **Table 7** | **Search strategy in CNKI (China National Knowledge Infrastructure)** |
| --- | --- |
| 专业检索  (期刊论文和学位论文) | SU=('肌少症'+肌肉减少症+少肌症+肌肉衰减综合症) AND SU=(运动+锻炼+体力活动+弹力绳+瑜伽+太极+气功+有氧锻炼+抗阻运动+久坐干预+运动干预) AND SU=(养老院+疗养院+老人院+照料中心+老年人） |

**Translated version**

| **Table 7** | **Search strategy in CNKI (China National Knowledge Infrastructure)** |
| --- | --- |
| Professional search  (journal studies and thesis) | (SU=(sarcopenia + sarcopenic + muscle wasting)) AND (SU=exercise + training+ sport + physical activity + resistance bands + yoga + Taiji + qigong + aerobic training + resistance training + sedentary behaviour intervention + physical inactivity intervention) AND (SU=nursing home + care homes + long-term care facilities + elderly + older adults) |

| **Table 8** | **Search strategy in Wanfang database** |
| --- | --- |
| 专业检索  （主题词扩展） | 题名或关键词:(肌少症 or 肌肉减少症 or 少肌症 or 肌肉衰减综合症) and 题名或关键词:(运动 or 锻炼 or 体力活动 or 弹力绳 or 瑜伽 or 太极 or 气功 or 有氧锻炼 or 抗阻运动 or 久坐干预 or 运动干预) and 题名或关键词:(养老院 or 疗养院 or 老人院 or 照料中心 or 老年人) |

**Translated version**

| **Table 8** | **Search strategy in Wanfang database** |
| --- | --- |
| Professional search (subject word expansion) | (Title or keyword: (sarcopenia or sarcopenic or muscle wasting) and (Title or keyword: (exercise or training or physical activity or sport or resistance bands or yoga or Taiji or qigong or aerobic training or resistance training or sedentary behaviour intervention or physical inactivity intervention) and (Title or keyword: (nursing home or care homes or long-term care facilities or elderly or older adults |
